# The transcriptional response of *Arabidopsis* leaves to Fe deficiency

**DOI:** 10.3389/fpls.2013.00276

**Published:** 2013-07-23

**Authors:** Jorge Rodríguez-Celma, I Chun Pan, Wenfeng Li, Ping Lan, Thomas J. Buckhout, Wolfgang Schmidt

**Affiliations:** ^1^Academia Sinica, Institute of Plant and Microbial BiologyTaipei, Taiwan; ^2^Institute of Biology, Humboldt University BerlinBerlin, Germany; ^3^Biotechnology Center, National Chung-Hsing UniversityTaichung, Taiwan; ^4^Genome and Systems Biology Degree Program, College of Life Science, National Taiwan UniversityTaipei, Taiwan

**Keywords:** Fe deficiency, chlorophyll metabolism, ribosomes, Fe homeostasis, reactive oxygen species, RNA-seq

## Abstract

Due to its ease to donate or accept electrons, iron (Fe) plays a crucial role in respiration and metabolism, including tetrapyrrole synthesis, in virtually all organisms. In plants, Fe is a component of the photosystems and thus essential for photosynthesis. Fe deficiency compromises chlorophyll (Chl) synthesis, leading to interveinal chlorosis in developing leaves and decreased photosynthetic activity. To gain insights into the responses of photosynthetically active cells to Fe deficiency, we conducted transcriptional profiling experiments on leaves from Fe-sufficient and Fe-deficient plants using the RNA-seq technology. As anticipated, genes associated with photosynthesis and tetrapyrrole metabolism were dramatically down-regulated by Fe deficiency. A sophisticated response comprising the down-regulation of *HEMA1* and *NYC1*, which catalyze the first committed step in tetrapyrrole biosynthesis and the conversion of Chl *b* to Chl *a* at the commencement of Chl breakdown, respectively, and the up-regulation of *CGLD27*, which is conserved in plastid-containing organisms and putatively involved in xanthophyll biosynthesis, indicates a carefully orchestrated balance of potentially toxic tetrapyrrole intermediates and functional end products to avoid photo-oxidative damage. Comparing the responses to Fe deficiency in leaves to that in roots confirmed subgroup 1b bHLH transcription factors and POPEYE/BRUTUS as important regulators of Fe homeostasis in both leaf and root cells, and indicated six novel players with putative roles in Fe homeostasis that were highly expressed in leaves and roots and greatly induced by Fe deficiency. The data further revealed down-regulation of organ-specific subsets of genes encoding ribosomal proteins, which may be indicative of a change in ribosomal composition that could bias translation. It is concluded that Fe deficiency causes a massive reorganization of plastid activity, which is adjusting leaf function to the availability of Fe.

## Introduction

In leaves, the vast majority of Fe is associated with the chloroplast, serving as a cofactor in all three photosynthetic electron transfer complexes. Iron deficiency is manifested in the interveinal chlorosis of developing leaves, which has been described more than one-hundred years ago as a diagnostic symptom of Fe deficiency (Gris, 1844). Chlorosis is caused by compromised chloroplast development and impaired chlorophyll (Chl) biosynthesis and is associated with dramatically decreased photosynthetic rates (Terry, 1980). The first step in tetrapyrrole biosynthesis is the formation of 5-aminolevulinic acid (ALA). Synthesis of ALA is regarded as the rate-limiting step in tetrapyrrole synthesis, and the glutamyl-tRNA reductase is the point of regulation (Tanaka et al., 2011). Interrupting the Chl biosynthesis pathway is detrimental to the plant due to severe oxidative damage caused by the generation of singlet oxygen by free tetrapyrroles upon illumination (op den Camp et al., 2003). A sensitive Fe sensing system associated with chloroplast function would allow plants to anticipate the inability to complete tetrapyrrole synthesis, to shut off the first committed step in the pathway and thus to avoid potentially detrimental consequences.

How Fe is sensed by plants is not known. In *Arabidopsis* roots, the earliest hub of the Fe signaling cascade is the bHLH protein FER-LIKE IRON DEFICIENCY INDUCED TRANSCRIPTION FACTOR (FIT), which positively controls a set of genes that are involved in the acquisition and distribution of Fe. Among them are the key genes that mediate rhiszosphere acidification (AHA2; Santi and Schmidt, 2009), the solubilization of un-available Fe pools (*F6'H1* and *PDR9*; Rodríguez-Celma et al., 2013), the reduction of Fe(III) chelates (*FRO2*; Robinson et al., 1999) and the uptake of the resulting Fe(II) (*IRT1*; Eide et al., 1996; Ling et al., 2002; Colangelo and Guerinot, 2004; Jakoby et al., 2004). FIT forms heterodimers with four Fe-responsive bHLH proteins, bHLH38, bHLH39, bHLH100, and bHLH101 (Yuan et al., 2005, 2008; Wang et al., 2012), presumably controlling different subsets of genes. Another bHLH transcription factor, POPEYE (PYE), negatively regulates a non-overlapping set of genes involved in Fe mobilization in roots and translocation of Fe to above-ground plant parts in association with the bHLH protein IAA-LEUCINE RESISTANT3 (ILR3/bHLH105) (Long et al., 2010). ILR3 also interacts with the putative DNA-binding E3 ubiquitin-protein ligase BRUTUS (BTS), which positively regulates the same set of genes. This dual regulation is thought to allow a fine-tuning of the expression of genes involved in Fe acquisition and cellular Fe homeostasis (Long et al., 2010).

Little information is available on putative candidates involved in sensing and signaling of Fe in chloroplasts or leaves. Sensing of the Fe status in the plastid would not only allow a close coupling of Chl synthesis, chloroplast development and photosynthesis rate, thus optimizing plant performance, but would also enable the plants to communicate the Fe status of the chloroplast to roots and to adjust Fe uptake to demand. Furthermore, the mechanisms that underlie Fe uptake in leaves have not been thoroughly described. Fe is transported to leaves as Fe^3+^-citrate chelate (Rellán-Álvarez et al., 2010), and might then be reduced either enzymatically by members of the FRO family or non-enzymatically by ascorbate. Based on their expression patterns and chlorotic mutant phenotypes, the YELLOW STRIPE-LIKE (YSL) transporters YSL1, YSL2, and YSL3 have been implicated in the uptake of Fe that has arrived in leaves via xylem transport (Didonato et al., 2004; Waters et al., 2006). Land plants evolved from chlorophyte algae, suggesting that conserved responses to Fe deficiency should be present in both groups. Indeed, a recent survey on transcriptional responses of *Chlamydomonas reinhardtii* revealed an overlap with Fe-responsive genes of *Arabidopsis thaliana*, including the metal transporters *IRT1, IRT2, NRAMP4*, and the P-type H^+^-ATPase *AHA2* (Urzica et al., 2012). However, the development of multicellularity in plants is accompanied by functional diversity of cells, tissues and organs. The contrasting roles of leaves and roots as sinks and sources for Fe are indicative of fundamentally different transcriptional responses in these organs.

To dissect the Fe deficiency response of leaf cells, we analyzed the changes in the transcriptome upon exposure to Fe deficiency in *Arabidopsis* leaves and compared this response with a previously published RNA-seq data set collected following the same treatment. The analysis revealed a suite of genes functioning in photosynthesis, chloroplast development and Chl synthesis that was rapidly and robustly regulated by Fe in leaves, indicating that the control of plastid function is a conserved and integral component of the *Arabidopsis* response to Fe deficiency. Our analysis further suggests that the composition of ribosomes by Fe is affected in an organ-specific manner, probably biasing translation. Lastly, we show that a small subset of highly expressed genes is robustly regulated by Fe deficiency in both roots and leaves, suggesting putative roles of the encoded proteins in Fe metabolism and the regulation of Fe homeostasis.

## Materials and methods

### Plant growth conditions

*Arabidopsis thaliana* plants were grown in a growth chamber on an agar-based medium as described by Estelle and Somerville (1987). Seeds of the accession Columbia (Col-0) were obtained from the Arabidopsis Biological Resource Center (Ohio State University). Seeds were surface-sterilized by immersing them in 5% (v/v) NaOCl for 5 min and 70% ethanol for 7 min, followed by four rinses in sterile water. Seeds were placed onto Petri dishes and kept for 1 d at 4°C in the dark, before the plates were transferred to a growth chamber and grown at 21°C under continuous illumination (50 μmol m^−2^ s^−1^; Phillips TL lamps). The medium was composed of (mM): KNO_3_ (5), MgSO_4_ (2), Ca(NO_3_)_2_ (2), KH_2_PO_4_ (2.5) and (μM): H_3_BO_3_ (70), MnCl_2_ (14), ZnSO_4_ (1), CuSO_4_ (0.5), NaCl (10), Na_2_MoO_4_ (0.2), FeEDTA (40), 4.7 mM MES, supplemented with sucrose (43 mM) and solidified with 0.4% Gelrite pure (Kelco). The pH was adjusted to 5.5.

For RNA-seq analysis, plants were pre-cultivated for 10 d in a complete medium, and then transferred to fresh agar medium either with 40 μM Fe(III)-EDTA (+Fe plants) or without Fe and with 100 μM 3-(2-pyridyl)-5,6-diphenyl-1,2,4-triazine sulfonate (ferrozine; −Fe plants) to trap residual Fe. Plants were grown for 3 d on Fe-free media before analysis. Three independent experiments were conducted for each growth type. For RT-PCR analysis, 10-day-old plants were transferred to hydroponic solution (Buckhout et al., 2009) for 3 d and then transferred to Fe-free solution for 15, 30, and 45 min, or for 1, 2, 4, and 6 h.

### RNA-seq

For RNA-seq, total RNA was extracted from the leaves and roots using the RNeasy Plant Mini Kit (Qiagen), following the manufacturer's instructions. For analysis, equal amounts of total RNA were collected and cDNA libraries for sequencing were prepared from total RNA following the manufacturer's protocol (Illumina). The cDNA libraries were subsequently enriched by PCR amplification. The resulting cDNA libraries were subjected to sequencing on a single lane of an Illumina Genome Analyzer II. RNA-seq and data collection was done following the protocol of Mortazavi et al. (2008). The length of the cDNA library varied from 250 to 300 bp with a 5′-adapter of 20 bp and a 3′-adapter of 33 bp at both ends.

To quantify gene expression levels, 75-mers sequences were aligned to the genomic sequence annotated in TAIR10 using the BLAT program (Kent, 2002), and RPKM (Reads Per Kilobase of exon model per Million mapped reads) values were computed using RACKJ (Read Analysis & Comparison Kit in Java, http://rackj.sourceforge.net/) software. Only those genes whose expression level in RPKM was over the square root of the mean expression value of the whole dataset (~4.5 RPKM) were considered as relevant for further analyses. Differentially expressed genes were selected based on Student's *t*-test (*P* < 0.05) and delta RPKM changes bigger than the mean expression value of the whole dataset (~20 RPKM) and/or 2-fold change in expression level between treatments.

### Bioinformatics

For gene clustering, we used the MACCU software (http://maccu.sourceforge.net/) to build co-expression networks based on co-expression relationships with a Pearson's coefficient greater than or equal to 0.60. In order to capture the tissue-specific co-expression relationships, Pearson's coefficients were computed based on robust multi-array averaged array data derived from leaf- and root-specific experiments for each tissue downloaded from NASCArrays (http://affymetrix.arabidopsis.info/). Visualization of the networks was performed with the Cytoscape software version 2.8.2 (http://www.cytoscape.org/).

### RT-PCR

For RT-PCR, total RNA was extracted from the leaves using Trizol (Invitrogen) and DNase treated with the Turbo DNA-free Kit (Ambion) following the manufacturer's instructions. cDNA was synthesized using QuantiTect Reverse Transcription Kit (Qiagen) following the manufacturer's instructions. Real-time PCR was performed using Power SYBR Green PCR Master Mix (Applied Biosystems) on an Applied Biosystems 7500 Fast Real-Time PCR System with programs recommended by the manufacturer. Samples were normalized first to an endogenous reference *(AtEF1α)* and then the relative target gene was determined by performing a comparative ΔΔCt. The following primers were used: *AtEF1α* (At5g60390) fwd: GAGCCCAAGTTTTTGAAGA, rev: CTAACAGCGAAACGTCCCA, and *HEMA1* (At1g58290) fwd: GATCTCCTTCTTCCACATCGCAA, rev: CCGCCATTGAAACCCAAAATC.

## Results

**Figure 1:**
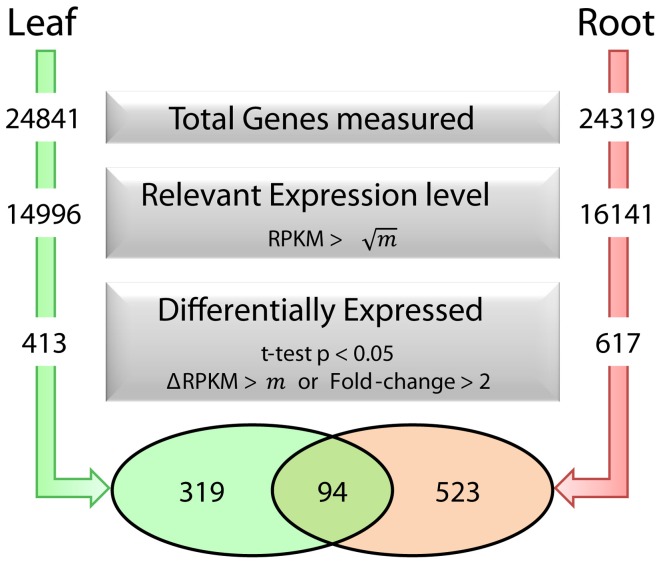
**Fe deficiency-induced transcriptional changes in leaves and roots and selection parameters used to identify differentially expressed genes.** *m* represents the average RPKM value for all genes detected in each dataset.

To dissect processes that are triggered by decreased Fe supply, we analyzed the transcriptome of Fe-sufficient and Fe-deficient leaves using the RNA-seq technology. A total of approximately 60 million reads was collected from three independent sequencing runs per growth type and aligned to the TAIR10 annotation of the *Arabidopsis* genome. A set of 413 genes was differentially expressed between Fe-deficient and Fe-sufficient plants (*t*-test *P* < 0.05 with either a FC > 2 or a ΔRPKM > 20.6), out of a total of 24841 genes detected in leaves from Fe-sufficient and Fe-deficient plants (Figure 1).

As anticipated from the high Fe demand of the photosynthetic electron chain, the strongest regulation was observed for photosynthesis-related genes. The expression of the PSI subunits *PSAF* and *PSAN* and of the ferredoxin *FED2* was decreased by more than 1000 RPKM; transcript levels of several other photosynthesis-associated genes such as the light harvesting complexes-related proteins *LHCB6, LHCA3, LHCA2*, and *LHCB4.1* were reduced by ca. 500 RPKM when grown on media lacking Fe. Furthermore, genes encoding Chl-binding proteins were strongly down-regulated. Interestingly, a subset of seven unknown proteins (At1g47400, At2g14247, At1g13609, At1g47395, At3g56360, At2g30766, and At5g67370) was most strongly up-regulated in leaves. In addition, the protein kinase *ORG1*, the oligopeptide transporter *OPT3* and the Ib subgroup transcription factors *bHLH38, bHLH39, bHLH100*, and *bHLH101* were strongly induced by Fe deficiency.

### Chlorophyll synthesis, Chl *b* to Chl *a* conversion and xanthophyll synthesis comprise a “photooxidative damage avoidance” module

**Figure 2:**
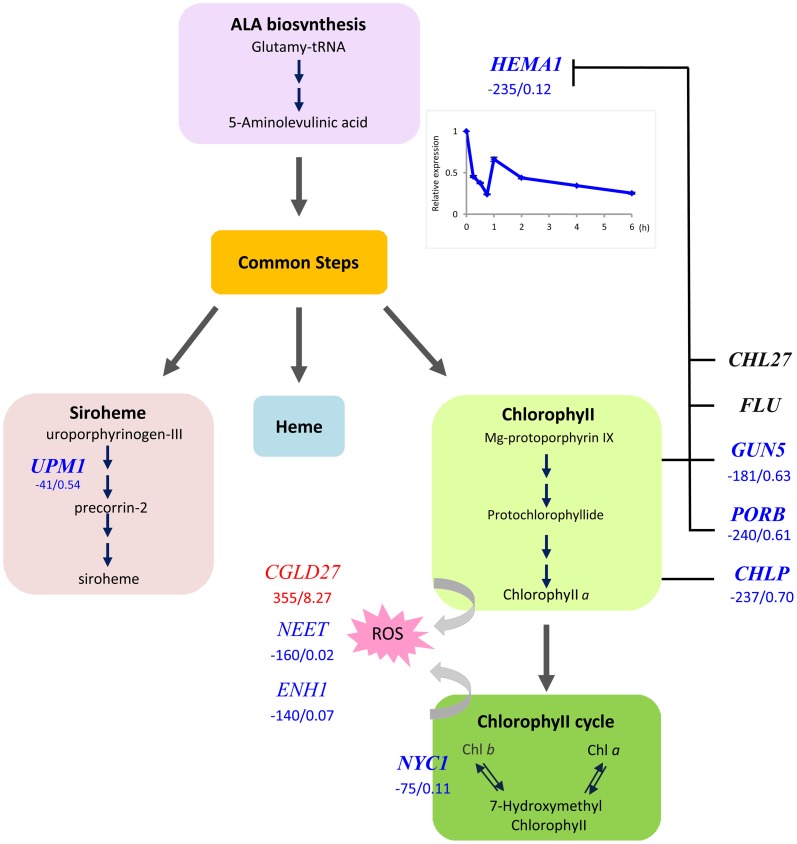
**Flow chart of the tetrapyrrole pathway toward siroheme, heme, and chlorophyll.** Enzymes that were transcriptionally affected by Fe deficiency are indicated by red (up-regulated genes) or blue letters (down-regulated genes). Numbers indicate delta RPKM and fold change of samples from Fe deficient compared with Fe-sufficient plants. Arrows denote steps that are likely regulated by the [Fe]. Inset shows the time-course of *HEMA1* expression in leaves after transfer to Fe-deplete media.

The universal symptom of Fe–deficient plants is interveinal chlorosis of developing leaves, which is the cause of the decreased Chl content. We found that the Glu-tRNA reductase (HEMA1), which catalizes the reduction of glutamyl-tRNA to glutamate-1-semialdehyde, the first committed step in tetrapyrrole biosynthesis, was massively down-regulated in Fe-deficient plants (Figure 2). The product of this first subdivision of Chl synthesis is 5-aminolevulinic acid (ALA). Down-regulation of *HEMA1* was robust and occurred rapidly after exposure of the plants to media lacking Fe. We observed a 2-fold decrease in transcript abundance in leaves of hydroponically grown plants after transfer of the plants to Fe-free media within 2 h (Figure 2, inset), suggesting a prompt adjustment of ALA biosynthesis to the available Fe. The second subdivision of the Chl biosynthesis pathway comprises six steps common to the heme, siroheme and Chl branches in which ALA is converted to protoporphyrin IX (Proto IX). None of the genes encoding enzymes mediating these common steps in tetrapyrrole biosynthesis was affected by Fe deficiency at the transcriptional level. Strong down-regulation occurred in three genes in the Chl branch, namely *GUN5, PORB*, and *CHLP* (Figure 2). This branch starts with the insertion of Mg^2+^ into Proto IX, mediated by magnesium chelatase (GUN5). PORB is one out of three *Arabidopsis* protochlorophyllide (Pchlide) oxidoreductases that catalize the reduction of the C17-C18 double bond in the D pyrrole ring to yield chlorophyllide *a*. *CHLP* encodes a geranylgeranyl reductase that catalyzes the reduction of geranylgeranyl pyrophosphate to phytyl pyrophosphate (Figure 2). A marked decrease in expression was also observed for *NON-YELLOW COLORING1* (*NYC1*), mediating the conversion of Chl *b* to Chl *a*. This conversion is important for Chl breakdown and is critical in light regulation (Tanaka et al., 2011). It can be assumed that the rate of Chl breakdown is adjusted to the decreased Chl synthesis under Fe-deficient conditions. Enzymes of the heme branch were not transcriptionally regulated by Fe. This is contrary to the anticipation since a lack of Fe will compromise the activity of ferrochelatases, inserting Fe^2+^ into proto IX to form protoheme. A weak down-regulation was observed for UPM1, catalyzing two steps in the siroheme branch.

*CONSERVED IN THE GREEN LINEAGE AND DIATOMS27* (*CGLD27*) belongs to a group of strongly up-regulated leaf genes. *CGLD27* has been described as one out of 14 Fe-responsive orthologs in *Chlamydomonas* and *Arabidopsis*, indicating that *CGLD27* is a common and potentially important component of the Fe deficiency response of the plant lineage (Urzica et al., 2012). *CGLD27 is* conserved in cyanobacteria and plastid-containing organisms but not in non-photosynthetic organisms, and CGLD27 likely targeted to plastids. Homozygous mutants are more sensitive to low Fe concentrations in the media (Urzica et al., 2012). The protein was predicted to function in carotenoid-xanthophyll metabolism (Kourmpetis et al., 2010) and is co-expressed with the ZETA-CAROTENE DESATURASE (ZDS; At3g04870) involved in the biosynthesis of carotenes and xanthophylls. It is tempting to speculate that CGLD27 has a function in photoprotection by quenching free radicals and singlet oxygen. Together the results indicate that HEMA1, NYC1, and CGLD27 may represent key players in preventing photooxidative damage in Fe-deficient leaf cells.

Two other genes encoding proteins that are targeted to chloroplast, AtNEET and NAP1 were strongly down-regulated in leaves from Fe-deficient plants. For both genes ancient roles in Fe metabolism were proposed (Xu et al., 2005; Nechushtai et al., 2012), indicating that these proteins may play a role in coordinating tetrapyrrole synthesis and ROS detoxification with the amount of available Fe.

### Co-expression networks from roots and shoots allows an integrative view of the *Arabidopsis* Fe deficiency response

**Figure 3:**
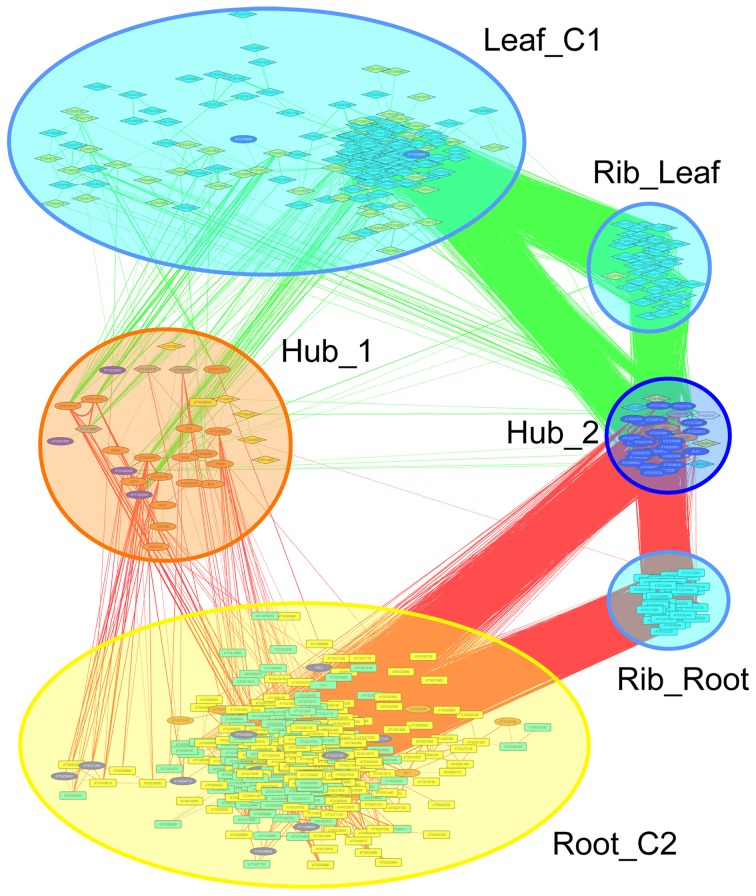
**Merged co-expression network of differentially expressed genes in roots and leaves.** Differentially expressed genes were first clustered separately for roots and shoots based on their co-expression relationships with a Pearson's coefficient of ≥0.60 against a customized data base that was constructed from experiments conducted with roots or leaves, respectively, using the MACCU software package, and then the two networks were combined. Yellow nodes denote genes that were up-regulated in roots or shoots, orange nodes indicate genes with increased expression in both organs. Light blue nodes denote genes that were down-regulated in roots or shoots, dark blue nodes indicate genes with decreased expression in both organs. Red and green lines represent co-expression edges derived from the root or leaf networks, respectively.

Co-expression networks can group genes to functionally related modules and can assign putative functions to unknown proteins. To identify modules with functions in the leaf response to Fe deficiency, we subjected the differentially expressed genes identified in our data set to a co-expression analysis using the MACCU software package (Lin et al., 2011). In an attempt to identify conserved and potentially co-regulated processes common to both leaves and roots, we compared transcriptional changes induced by growth on Fe depleted media in leaves with those of roots by re-analyzing a previously published data set of roots from plants grown under the same conditions (Rodríguez-Celma et al., 2013). In roots from Fe-deficient plants, 617 genes were found to be differentially expressed when similar criteria were applied (Figure 1). We then analyzed the co-expression of genes in leaves and roots separately using databases of publically available microarray experiments that discriminate for leaf- and root-related processes. Finally, we merged the two sub-networks, yielding a large hybrid network comprising 725 genes that can be subdivided in several subclusters (Figure 3, Table S1 and Figure S1). Two subclusters (Hub_1 and Hub_2) connect the two major clusters. The cluster Root_C2 contains genes involved in the acquisition of Fe and in regulation of Fe homeostasis. The largest changes in transcript abundance were observed for genes that mediate the acquisition of Fe (*IRT1* and *AHA2*), genes catalyzing the first steps in the general phenylpropanoid pathway (*F6'H1, PAL1, PAL2*), the detoxification of transition metals (*MTPA2*) and in the regulation of Fe homeostasis (*FIT*). Also, several genes with yet undefined function in Fe homeostasis were highly responsive to the change in growth regime. The most strongly down-regulated gene was *PYK10*, encoding a β-glucosidase located in the ER (Nitz et al., 2001). *PYK10* was suggested to restrict colonization of the beneficial endophytic fungus *Piriformospora indica*, thereby conferring resistance to heavy metal ions and promoting nutrient uptake (Sherameti et al., 2008). It can be assumed that lowered expression of *PYK10* compensates for reduced interaction between roots and the fungus due to decreased Fe content of root cells. While this assumption awaits further experimental support, the data might hint at an understudied aspect of the Fe-deficiency response that may be of importance for the fitness of plants under natural conditions.

**Figure 4:**
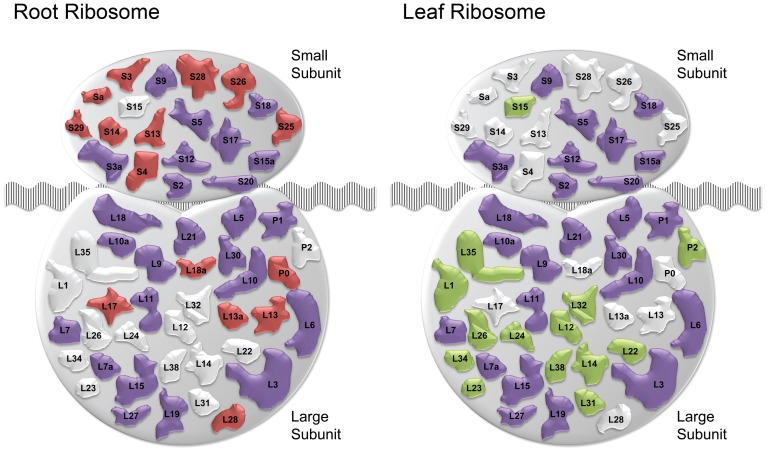
**Fe deficiency-induced changes in ribosomal composition.** Schematic of ribosomal composition changes for roots and leaves. Red and green shapes represent ribosomal protein families down-regulated specifically in roots and leaves, respectively. Purple colored shapes represent families down-regulated in both organs. White colored shapes denote families not regulated in the corresponding tissue ribosomes.

The cluster Leaf_C1 contained several genes associated with PS, LHCs and with Chl synthesis that have been described above. Genes derived from the root and shoot network were connected by three relatively large subclusters containing ribosome genes. Notably, two of the ribosomal subclusters (Rib_Leaf and Rib_Root) were comprised of genes that were exclusively derived from the root and shoot network, and the third subcluster contained genes from both subnetworks. Inspection of the genes in these subclusters revealed that the down-regulation of genes encoding ribosomal proteins was differential between leaves or roots. Notably, in leaves proteins belonging to paralogs families of the large subunit were affected by Fe deficiency, while in roots the majority of regulated ribosomal genes encoded ribosomal proteins from the 40S subunit (Table S2). This suggests that the composition of ribosomes is changed by Fe deficiency and that this change occurs in an organ-specific manner (Figure 4).

### A conserved set of Fe-regulated genes is highly expressed in both leaves and roots

Using conservative criteria (FC > 2, *P* < 0.05 and ΔRPKM > 40), we mined the data sets for genes that are strongly Fe-regulated in both leaves and roots. A set of 17 genes fulfilled these criteria, among them the transcription factors *bHLH38, bHLH39* and *bHLH100, FRO3, OPT3*, the nicotianamine synthase *NAS4*, the Fe storage protein *FER1*, the Fe superoxide dismutase *FDS1* and the protein kinase *ORG1* (Table 1), some of which were reported to be Fe-regulated in previous microarray experiments (Ivanov et al., 2011). Furthermore, six proteins in this set have unknown functions (Table 1). The high expression levels and the robust regulation of the genes encoding these proteins indicate putatively important functions in Fe metabolism. We have named them IRON-RESPONSIVE PROTEIN (IRP) 1–6, as candidates for follow-up experiments (Table 1). The encoded proteins can be organized in three groups of two proteins. IRP1 (At1g47400) and IRP2 (At1g47395) are peptides of 50 amino acids with a conserved region. The second group comprises IRP3 (At2g14247) and IRP4 (At1g13609), small proteins of 78 amino acids and no conserved domain. IRP5 (At3g56360) and IRP6 (At5g05250) are 25.1 and 25.9 kDa proteins, respectively, with predicted myristoylation sites.

**Table 1:** **Genes that were differentially expressed between Fe-sufficient and Fe-deficient plants in both leaves and roots with a ΔRPKM > 40 and FC > 2 at *P* < 0.05**.

**ID**	**Symbol**	**Description**	**Leaf ΔRPKM**	**Leaf FC (log_2_)**	**Root ΔRPKM**	**Root FC (log_2_)**
At1g47400	IRP1	Iron-responsive protein 1	397.47	6.44	63.29	4.11
At1g47395	IRP2	Iron-responsive protein 2	797.71	6.30	139.65	3.89
At2g14247	IRP3	Iron-responsive protein 3	432.04	6.30	142.85	2.37
At1g13609	IRP4	Iron-responsive protein 4	682.77	7.97	80.11	6.27
At3g56360	IRP5	Iron-responsive protein 5	318.11	2.04	82.54	1.24
At5g05250	IRP6	Iron-responsive protein 6	238.88	4.04	58.06	2.72
At3g56970	bHLH38	Basic helix-loop-helix protein 38	275.66	10.17	144.41	6.19
At3g56980	bHLH39	Basic helix-loop-helix protein 39	154.59	9.45	120.43	4.96
At2g41240	BHLH100	Basic helix-loop-helix protein 100	363.10	12.12	105.43	8.14
At1g23020	FRO3	Ferric reduction oxidase 3	124.84	2.55	87.69	2.91
At4g16370	OPT3	Oligopeptide transporter 3	300.81	2.49	80.60	2.77
At5g53450	ORG1	OBP3-responsive gene 1	390.12	4.39	57.89	2.39
At1g56430	NAS4	Nicotianamine synthase 4	92.59	2.70	43.08	2.39
At4g08390	SAPX	Stromal ascorbate peroxidase	−98.62	−3.41	−50.32	−1.06
At4g25100	FSD1	Fe superoxide dismutase 1	−239.22	−4.26	−57.45	−2.15
At5g01600	FER1	Ferritin 1	−233.03	−4.85	−60.79	−2.29
At1g48300	DGAT3	Diacylglycerol acyltransferase 3	260.16	1.65	143.56	1.18

**Figure 5:**
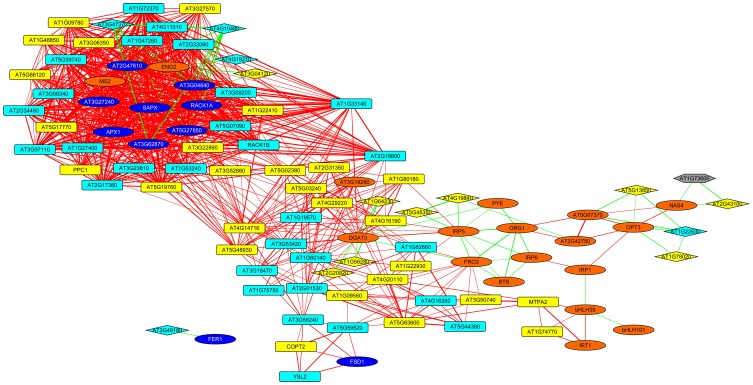
**Co-expression network around genes that are differentially expressed in roots and leaves.** The genes listed in Table 1 were used as baits and first-degree neighbors were added based on a database comprising publicly available microarray experiments. Yellow nodes denote genes that were up-regulated in roots or shoots, orange nodes indicate genes with increased expression in both organs. Light blue nodes denote genes that were down-regulated in roots or shoots, dark blue nodes indicate genes with decreased expression in both organs. Red and green lines represent co-expression edges derived from the root or leaf networks, respectively. Squared and diamond shaped nodes represent genes differentially expressed in roots and leaves, respectively.

To put the conserved genes into a functional context and to assign putative functions for the unknown proteins, we constructed a co-expression network using the set of 17 conserved genes as baits and fished for first degree co-expressed nodes (Figure 5). The resulting network was comprised 99 genes with several central players in Fe homeostasis such as *OPT3, FRO3, NAS4, IRT1, MTPA2*, and the phosphoenolpyruvate carboxylase *PPC1*. Also, four major regulators of Fe homeostasis were found within this network, including *BTS, PYE, bHLH39*, and *bHLH101*. Notably, with the exception of *IRT1, MTPA2*, and *PPC1*, which appear more important for root Fe acquisition, all the genes listed above plus *HEMA1* and *FER4* were located in HUB_1 (Figure 3) that connects the genes that are specific for roots or leaves, indicating a function of these genes in both organs and/or in systemic Fe homeostatic regulation. This network also contained 16 genes encoding ribosomal proteins and two orthologs of the mammalian receptor for activated C-kinase 1 (*RACK1*), *RACK1A*, and *RACK1B*. In both mammals and plants, *RACK1* genes have been associated with protein translation and ribosome biogenesis (Nilsson et al., 2004; Guo et al., 2011). Together these results indicate conserved players in cellular Fe homeostasis. They establish a link between several novel genes encoding proteins with unknown functions and a regulatory network.

## Discussion

While the transcriptional response of roots to Fe deficiency is well explored at the whole-genome level, information on transcriptional profile changes that occur in Fe-deficient leaves is scarce. The largest sinks for Fe are PSI and PSII, and the major fraction of Fe is located in the chloroplasts. It seems reasonable to assume that photosynthetic cells have evolved mechanisms that adjust the synthesis of light harvesting complexes to that of Fe-containing enzymes and complexes to avoid accumulation of ROS and to recalibrate Fe homeostasis in leaf cells.

### Photooxidative damage in Fe-deficient leaf cells is avoided by elaborate mechanisms

Decreased Chl content is a hallmark of Fe-deficient plants and has been associated with compromised photosynthesis caused by decreased tetrapyrrole biosynthesis. The fast and pronounced down-regulation of *HEMA1* expression suggests a rapid and dramatic decline of tetrapyrrole synthesis at an early stage of Fe deficiency. Our data support a scenario in which plastid Fe sensing links Chl synthesis to the activity of the photosystems to avoid the production of toxic radicals upon illumination of free tetrapyrrole intermediates. Notably, Fe-deficient *Clamydomonas* cells are not sensitive to high light and photosynthetic mutants grow better on low Fe media (Moseley et al., 2002), demonstrating that Fe-deficient plants are protected against photo-oxidative damage. ALA synthesis is rate-limiting in tetrapyrrole synthesis and sophisticatedly regulated (Tanaka et al., 2011; Brzezowski and Grimm, 2013). Interestingly, in acetate-grown *Chlamydomonas* cells tetrapyrrole synthesis was up-regulated upon Fe deficiency, while photoautotrophic cells showed lower mRNA levels of the enzymes involved in the pathway (Moseley et al., 2002), suggesting that Chl synthesis is not restricted by decreased Fe supply. Similar to other regulators of tetrapyrrole synthesis such as light, phytohormones and the circadian clock, the control sites with regard to Fe availability are at the beginning, at the branch points and at the end of the pathway. This avoids the accumulation of photoreactive porphyrin and of free Chl intermediates, and adjusts the metabolic flow to demand. The metabolites Mg-protoporphyrin and divinylprotochlorophylide *a* negatively regulate HEMA1 activity (Tanaka and Tanaka, 2007). Recently, it has been shown that in the presence of Pchlide a complex comprising the fluorescence protein FLU, PORB, CHL27, and CHLP interacts with HEMA1 and inhibits ALA synthesis (Figure 2; Kauss et al., 2012). In the absence of Pchlide, such interaction is compromised and allows ALA synthesis (Kauss et al., 2012). How are Chl synthesis and photosynthetic activity synchronized to avoid accumulation of ROS in Fe-deficient leaves? A possible scenario involves a Chl-mediated disconnection of the LHC1 antenna from PSI as described for *Chlamydomonas* (Moseley et al., 2002). A finely tuned Chl synthesis and binding of Chl to PSI would allow an efficient adaptation of the photosynthetic machinery to the available Fe pools. The diiron Mg-protoporphyrin IX monomethylester oxidative cyclase CHL27 has been suggested as a candidate plastid Fe sensor (Tottey et al., 2003). Another potential candidate that links photosynthesis, ROS and Fe metabolism is the NEET protein At5g51720 (AtNEET). AtNEET harbors two redox active 2Fe-2S clusters and is dramatically down-regulated (40-fold; 98% decrease in transcript level) in leaves of Fe-deficient plants. *AtNEET* is tightly co-expressed with *FER3* and *FER4*, encoding plastid ferritins. Furthermore, *AtNEET* is co-expressed with *ENH1*, which may play a role in ROS detoxification (Zhu et al., 2007). Reduced levels of *AtNEET* transcript levels caused late greening, increased the sensitivity to low Fe levels, and conferred insensitivity to high Fe concentrations (Nechushtai et al., 2012). NEET proteins are conserved in plants and mammals and an ancient role of AtNEET in Fe homeostasis has been suggested (Nechushtai et al., 2012). However, a clear role in plastid Fe metabolism has not yet been defined. Another candidate for a plastid Fe sensor is the NON-INTRINSIC ABC PROTEIN (NAP1), a protein with similarities to prokaryotic *SufB* proteins which are involved in Fe-S cluster repair (Xu et al., 2005). AtNAP1 complements an *E. coli SufB* mutant, indicating that AtNAP1 is an evolutionary conserved SufB protein (Xu et al., 2005), and regulation of cellular sulfur level has been previously shown to be related to Fe homeostasis (Ivanov et al., 2011). AtNAP1 is transcriptionally and post-transcrioptionally regulated by Fe, and may function in plastid Fe homeostasis (Xu et al., 2005). A strong down-regulation of *AtNAP1* was observed in the present study (Table S1), supporting the assumption of a role of AtNAP1 in plastid Fe sensing or signaling.

### Fe deficiency alters ribosome composition in an organ-specific manner

While considered as housekeeping genes encoding structural components, plant cytosolic ribosomes are more heterogenous and plastic than their mammalian counterparts. Plant ribosomal proteins are encoded by multiple divergent paralogs, which allows, in theory, for 10^34^ structurally different ribosomes (Hummel et al., 2012), a number that can be increased by numerous post-translational modifications. Generally, ribosome biogenesis appears to be down-regulated in both roots and leaves from Fe-deficient plants. A large part of transcriptional activity is used for this process and the decrease in the abundance of ribosomal subunits may be interpreted in terms of biasing translation toward proteins encoding products that are needed in large amounts to recalibrate cellular Fe homeostasis. It also appears, however, that the down-regulation is not general but highly organ-specific, indicating a carefully controlled change in ribosomal composition. Paralog composition has also been shown to be altered by growth conditions (Hummel et al., 2012), which is most likely the case in Fe-deficient plants. The fact that the change in the expression of paralogous isoforms is typical of leaves and roots suggests that this change is not due to a general down-regulation of translation, but rather may adjust the protein composition of the cell to the prevailing conditions. This would also offer an explanation for the relatively low concordance of proteomic and transcriptomic changes in response to environmental conditions (Lan et al., 2012). Importantly, our results indicate that translational bias, mediated by a change in composition of ribosomes, is part of the Fe deficiency response of both leaves and roots.

### Established and novel players control cellular Fe homeostasis in leaves and roots

Despite the different function of leaf and root cells, many of the major regulators of Fe deficiency responses that have been identified in roots were also found in leaves, indicating that the subgroup 1b transcription factors and PYE/BTS are controlling cellular Fe homeostasis in most if not all plant cells (Ivanov et al., 2011). A notable exception was FIT, the expression of which appears to be root-specific. It seems plausible that different combinations of heterodimers are regulating non-overlapping sets of genes, a scenario which does not exclude other levels of regulation. A novel finding was the differential expression of ribosomal proteins, which possibly altered the composition of ribosomes in an organ and growth type-specific manner, a regulatory layer which has yet to be explored. Our data also revealed several new players that may play conserved roles in Fe metabolism in both leaves and roots. Some of these genes have no corresponding probe set on the ATH1 gene chip and can thus not be properly inserted into co-expression networks. The three genes for which a co-expression relationship can be inferred from public data bases (*IRP1, IRP5*, and *IRP6*), are closely co-expressed with each other and several genes with important functions in Fe homeostasis (e.g., *NRAMP4, FRO3, NAS4, bHLH39, OPT3, ORG1*, and *PYE*), associating putative roles in Fe metabolism of these proteins in both leaves and roots.

No transporter from the YLS, ZIP, or IREG family that could mediate the transport of Fe(II) has been found to be Fe-regulated in leaf cells. Such a function could be fulfilled by OPT3, as inferred from the strong phenotype of the *opt3-2* mutant (Stacey et al., 2008), the general characteristics and expression patterns of OPT transporters (Lubkowitz, 2011), the massive up-regulation of the genes upon Fe deficiency and the central position in the co-expression networks.

Several lines of evidence support a concept of anticipated changes that have been proven for the sequestering of surplus metals that are associated with an up-regulation of IRT1 activity (Yang et al., 2010). For example, down-regulation of *PYK10* can be interpreted as an anticipation of a Fe deficiency-induced decrease in colonization efficiency of *P. indica*. Also, the fast orchestrated regulation of tetrapyrrole synthesis, Chl *b* to a Chl *a* conversion and, putatively, the biosynthesis of xanthophyll to avoid oxidative damage may be interpreted as anticipatory changes. Understanding of the regulation of these response modules is desirable in order to gain a holistic understanding of the responses to Fe deficiency and also in order to develop tools to generate plants with improved Fe efficiency.

### Conflict of interest statement

The authors declare that the research was conducted in the absence of any commercial or financial relationships that could be construed as a potential conflict of interest.
