# Supplementary figures and images for: The transcriptional response of Arabidopsis leaves to Fe deficiency
Source: Front Plant Sci. 2013 Jul 23;4:276. doi: 10.3389/fpls.2013.00276 (PMC3719017; doi:10.3389/fpls.2013.00276)

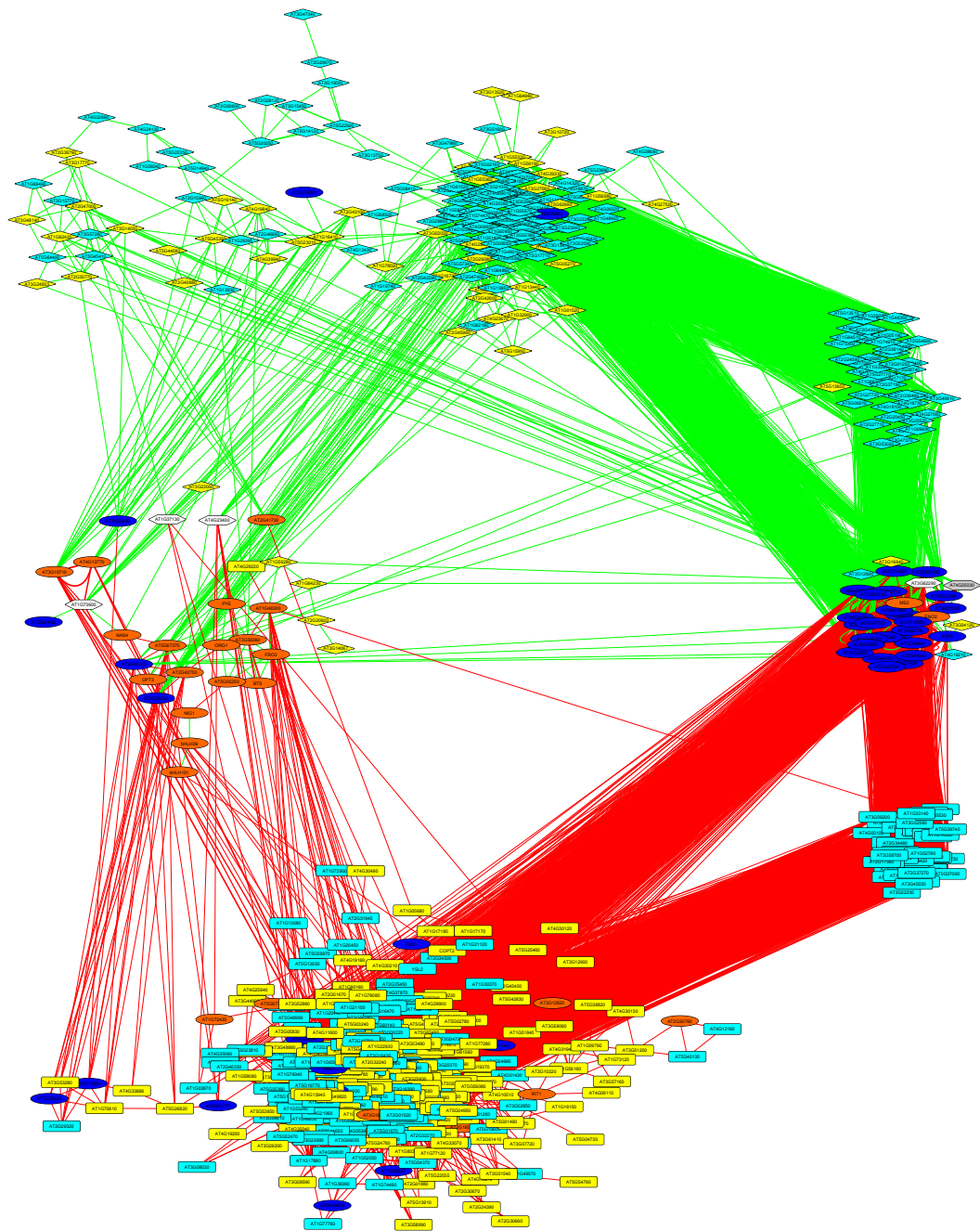

Supplement: Supplementary file 3 [file DataSheet3.PDF]
